# Supplementary material for: Brief, Repeated Isoflurane Anaesthesia Elicits Sex‐Dependent Behavioural, Cerebrovascular and Microglial Alterations in Mice
Source: Eur J Neurosci. 2026 Aug 26;64(4):e70663. doi: 10.1111/ejn.70663 (PMC13519372; doi:10.1111/ejn.70663)
Supplement: Supplementary file 1 [file EJN-64-0-s001.pdf]

Supplemental Figure 1: Gating strategy for identification of primary microglia (CD11b+ve CD45low)

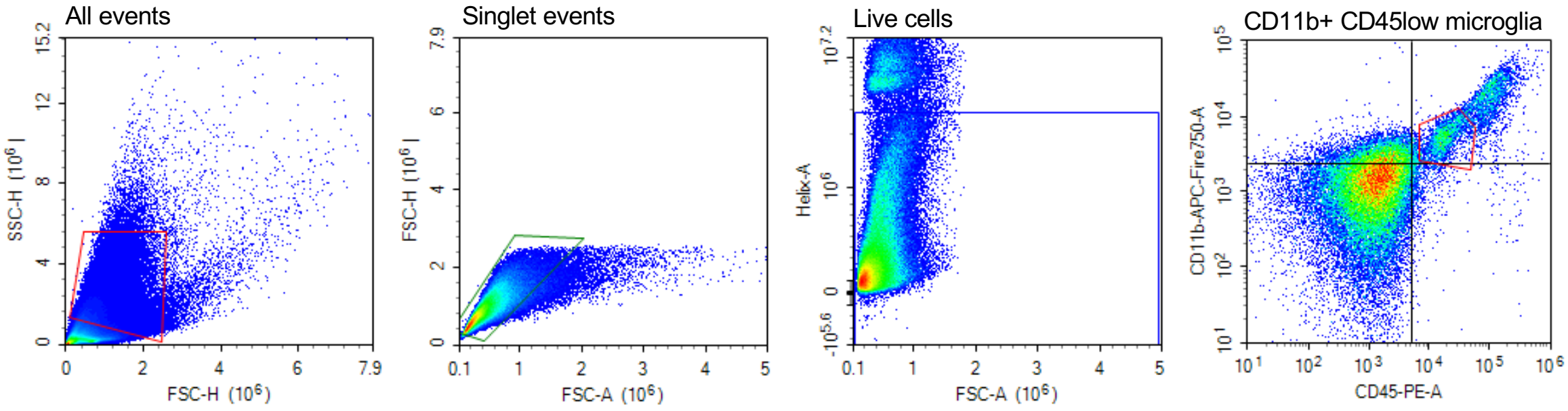

Supplemental Figure 2: Comparison of circadian rhythm parameters between male and female mice at baseline and following six periods of isoflurane anaesthesia. A) Typical diurnal and nocturnal activity plots for individual mice at baseline and after the sixth round of anaesthesia (ISO6). B) Male mice had a significantly longer acrophase (time between lights on and maximum activity) than female mice, but this was not affected by isoflurane exposure, data are mean ± SEM, n=6; C) The circadian cycle amplitude (the difference between the peak and trough of activity) is suppressed by isoflurane exposure in female mice, but increased in male animals, resulting in a loss of the baseline sex difference, data are mean ± SEM, n=6; D) Repeated isoflurane exposure has no effect on the nocturnality index, the proportion of activity occurring during the dark phase, in either male or female mice, data are mean ± SEM, n=6; E) Repeated isoflurane exposure reduces dark phase anticipation (activity in the two hours prior to lights off) in both male and female mice, data are mean ± SEM, n=6.

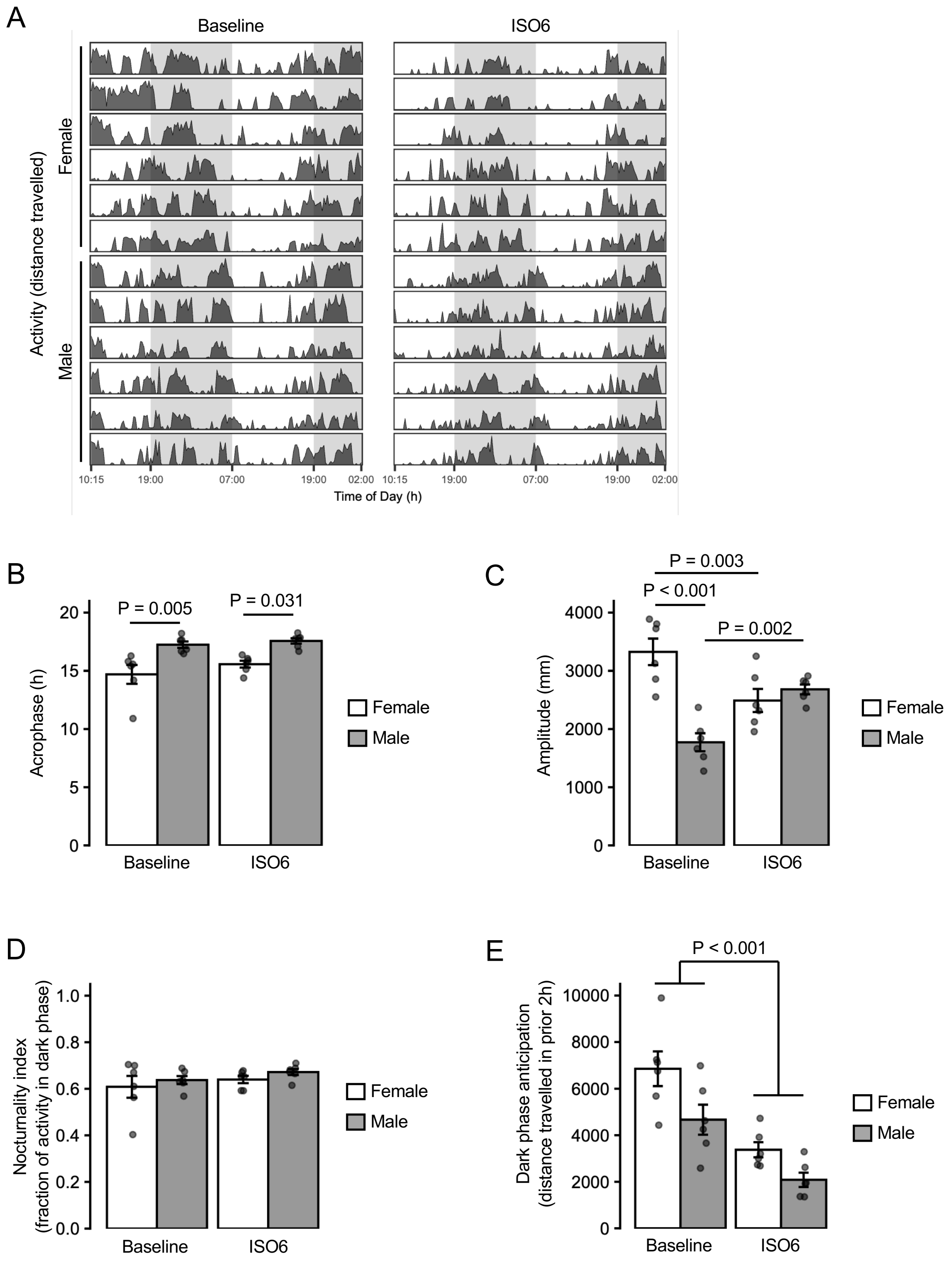

Supplemental Figure 3: Frequency of (A) grooming, (B) feeding, (C) drinking, (D) clustering, (E) brief rearing (<3s), (F) prolonged rearing (>3s), (G) food rearing, (H) climbing, (I) hanging, (J) scrape digging, (K) tube running, (L) chasing, (M) anogenital investigation, (N) aggression, and (O) allo-grooming behaviours per observation period during baseline and following each isoflurane induction session (ISO1–ISO6) for female (red) and male (blue) mice. Data are mean ± SEM, n=2–6 group-housed cages of 3 mice. Full descriptions of each behavioural category are provided in Supplemental Table 1.

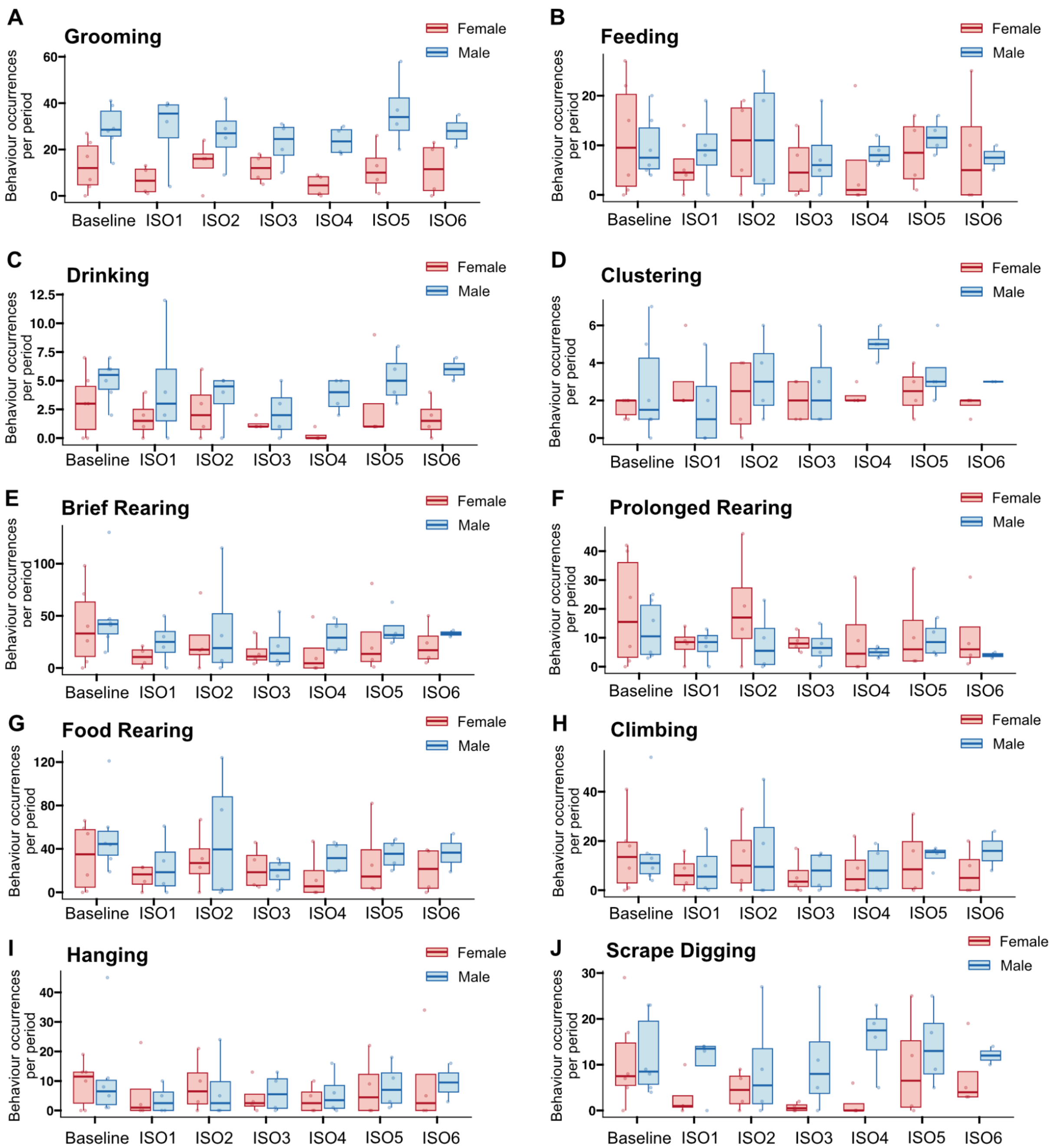

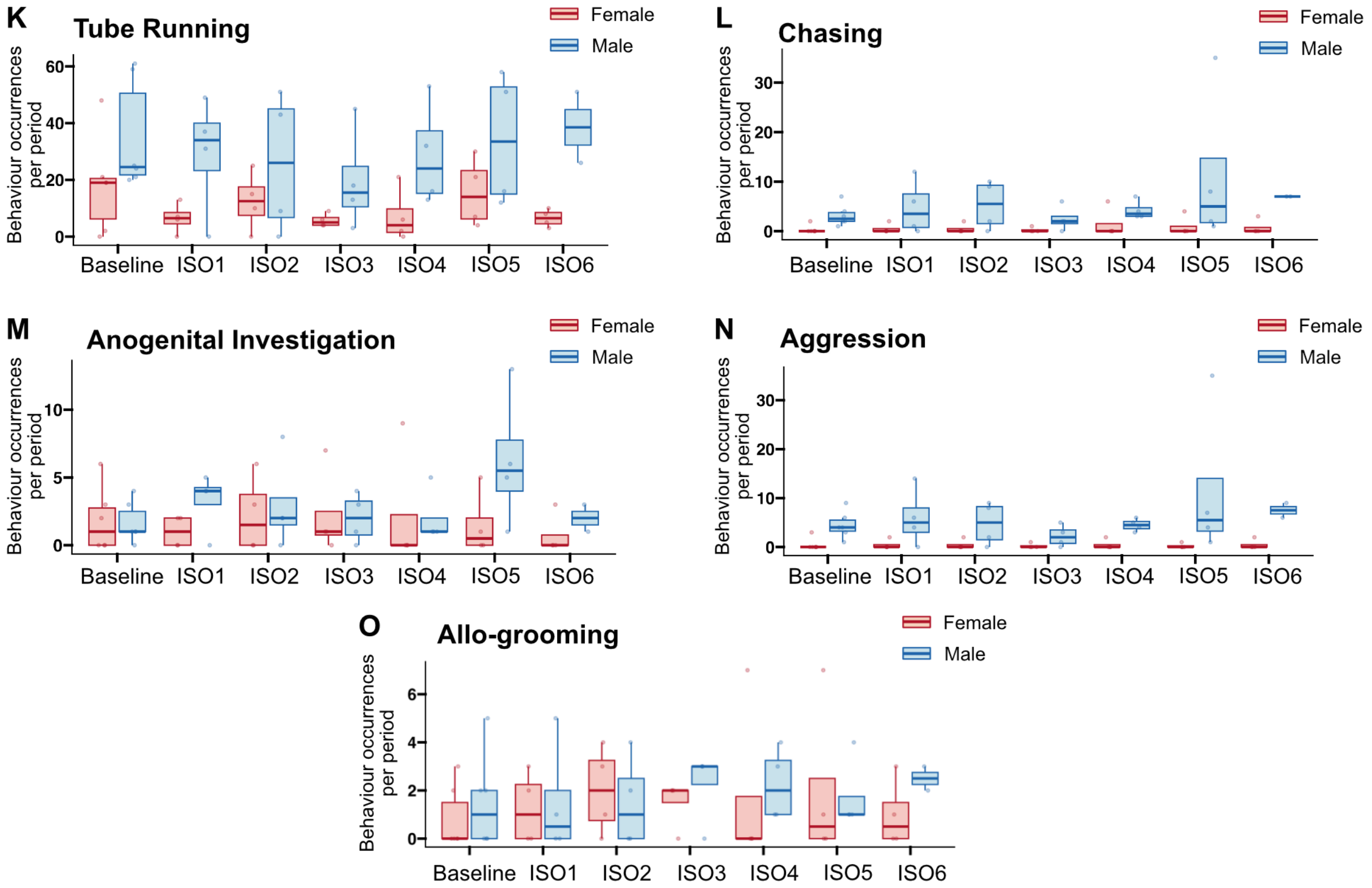
K
Tube Running
Female
Male
Behaviour occurrences per period
0
20
40
60
Baseline
ISO1
ISO2
ISO3
ISO4
ISO5
ISO6
L
Chasing
Female
Male
Behaviour occurrences per period
0
10
20
30
Baseline
ISO1
ISO2
ISO3
ISO4
ISO5
ISO6
M
Anogenital Investigation
Female
Male
Behaviour occurrences per period
0
5
10
Baseline
ISO1
ISO2
ISO3
ISO4
ISO5
ISO6
N
Aggression
Female
Male
Behaviour occurrences per period
0
10
20
30
Baseline
ISO1
ISO2
ISO3
ISO4
ISO5
ISO6
O
Allo-grooming
Female
Male
Behaviour occurrences per period
0
2
4
6
Baseline
ISO1
ISO2
ISO3
ISO4
ISO5
ISO6

Supplemental Table 1: Ethogram depicting typical murine behaviours analysed in infrared video recordings, grouped as maintenance, exploratory or social behaviours.

**Maintenance Behaviours**			**Exploratory Behaviours**		
Grooming	The mouse cleans its fur and body, licking its paws and then using them to groom the rest of the body.	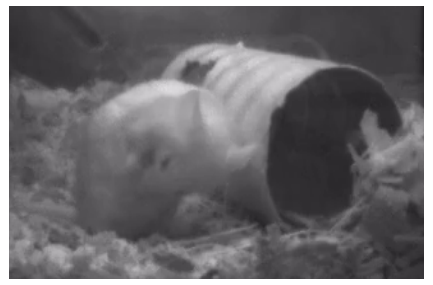	Brief rearing	The mouse briefly stands upright on its hindlimbs for ≤ 3 s, elevating its forelimbs and head. It may also stand against the wall in this posture.	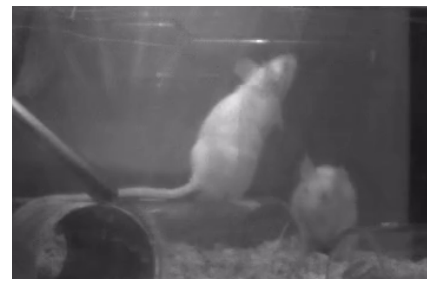
Nesting	The mouse uses provided materials to construct a nest, digging, pushing and carrying material around the cage.	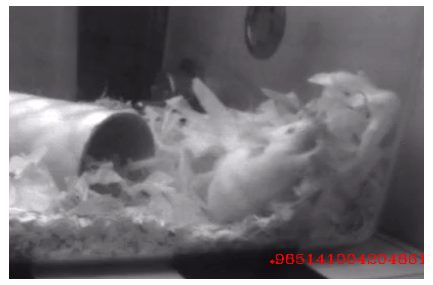	Prolonged rearing	Same posture as brief rearing, but lasting > 3 s.	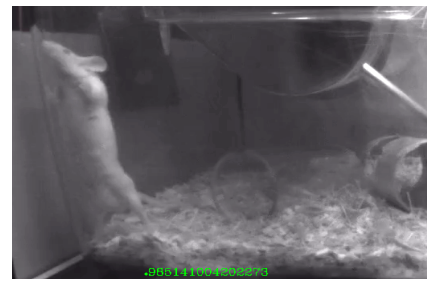
Burrowing	The mouse digs through a pile of nesting material to create a tunnel where it can rest in.	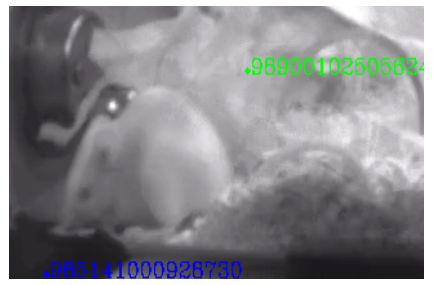	Food rearing	As with other rearing behaviours, but with the mouse positioning its forelimbs on the food dispenser.	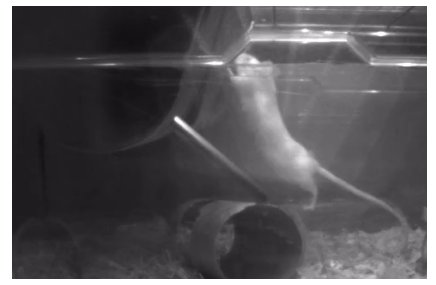
Feeding	The mouse eats food in the food dispenser using its forelimbs, or bites food pellets on the cage floor.	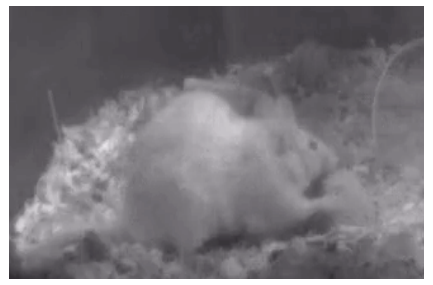	Climbing	The mouse hangs on the side of the food dispenser without contact with the ground.	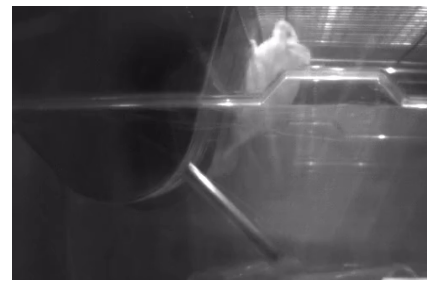
Drinking	The mouse licks the water dispenser to drink. It may use its forepaws to hold the spout.	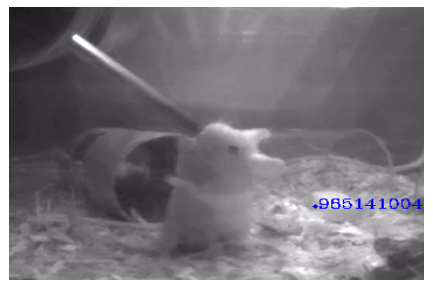	Hanging	The mouse hangs upside-down on the ceiling of the cage. The mouse moves around the ceiling area using its fore- and hindlimbs to grasp the cage bars.	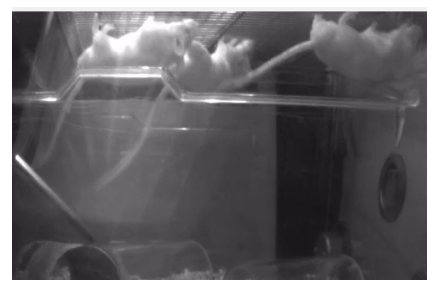
Clustering	Two or more mice huddle together, often observed during sleep.	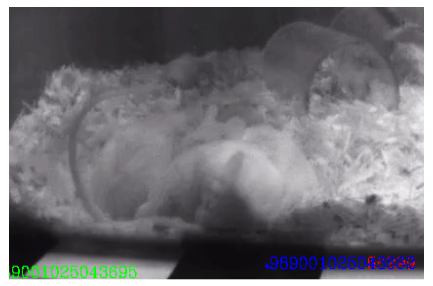	Scrape digging	The mouse vigorously digs into the cage nesting using both its fore- and hindlimbs.	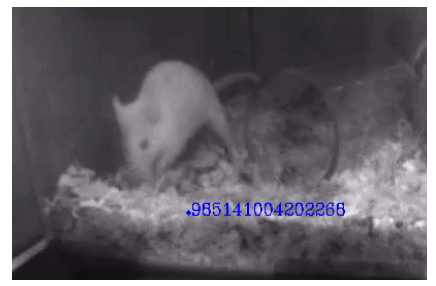
			Tube running	The mouse runs through a provided tube without stopping or turning around within the tube.	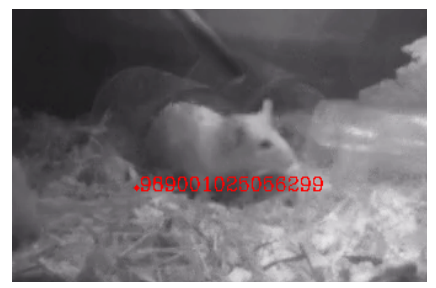

Social Behaviours		
Chasing	One mouse pursues another as it flees, an interaction that typically frequently ends in aggression from the chasing mouse.	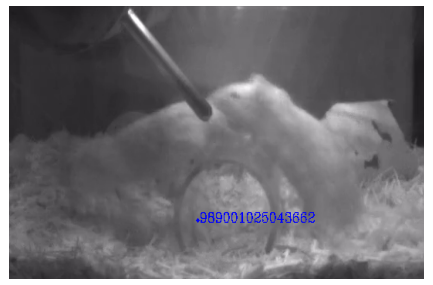
Anogenital investigation	One mouse positions its nose near the genital area of another mouse. This behaviour may be reciprocal or non-reciprocal.	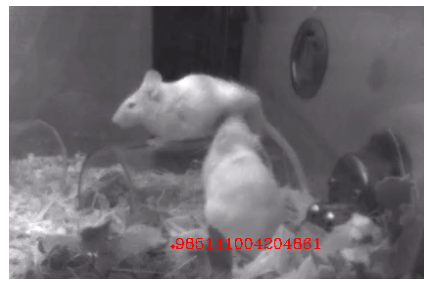
Aggression	One mouse attacks another, either unilaterally or with aggression on both parts. This includes behaviours such as biting, boxing, and attempted mating.	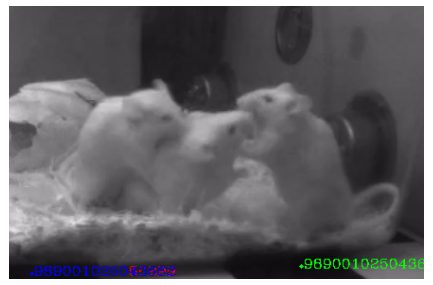
Allo-grooming	Similar to grooming, but involving one mouse grooming another.	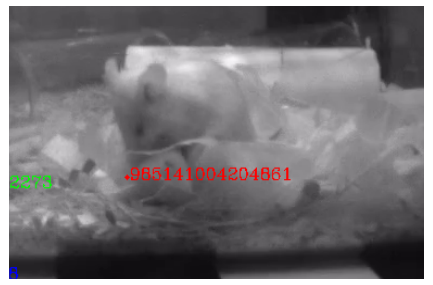

Supplemental Table 2: Statistical comparisons of ethogram data. Significant main/interaction effects are highlighted in bold text.

Measure	Treatment	Sex	Time of day	Treatment x Sex	Treatment x Time of day	Sex x Time	Treatment x Sex x Time of day
**Rearing <3s**	$F_{6,26}$ = 1.922, P=0.115	$F_{1,26}$ = 2.445, P=0.130	**$F_{1,26}$ = 7.96, P=0.009**	$F_{6,26}$ = 0.028, P=0.999	$F_{6,26}$=0.912, P=0.502	$F_{1,26}$=2.835 P=0.104	$F_{6,26}$=0.201 P=0.973
**Rearing ≥ 3s**	$F_{6,26}$ = 2.191, P=0.076	$F_{1,26}$ = 3.156, P=0.087	**$F_{1,26}$ = 17.03, P<0.001**	$F_{6,26}$ = 0.435, P=0.848	$F_{6,26}$=1.544, P=0.203	$F_{1,26}$=15.243 P<0.001	$F_{6,26}$=0.327 P=0.916
**Food rearing**	$F_{6,26}$ = 0.939, P=0.484	$F_{1,26}$ = 2.298, P=0.142	**$F_{1,26}$ = 11.12, P=0.003**	$F_{6,26}$ = 0.168, P=0.983	$F_{6,26}$=0.401, P=0.872	$F_{1,26}$=1.72 P=0.201	$F_{6,26}$=0.144 P=0.989
**Climbing**	$F_{6,26}$ = 1.001, P=0.445	$F_{1,26}$ = 596, P=0.446	**$F_{1,26}$ = 16.012, P<0.001**	$F_{6,26}$ = 0.062, P=0.998	$F_{6,26}$=0.589, P=0.736	$F_{1,26}$=1.722 P=0.201	$F_{6,26}$=0.138 P=0.99
**Hanging**	$F_{6,26}$ = 0.761, P=0.607	$F_{1,26}$ = 0.083, P=0.775	**$F_{1,26}$ = 15.743, P<0.001**	$F_{6,26}$ = 0.127, P=0.992	$F_{6,26}$=0.567, P=0.752	$F_{1,26}$=0.498 P=0.487	$F_{6,26}$=0.192 P=0.976
**Chasing**	$F_{6,26}$ = 0.652, P=0.688	**$F_{1,26}$ = 9.193, P=0.005**	$F_{1,26}$ = 0.950, P=0.339	$F_{6,26}$ = 0.547, P=0.767	$F_{6,26}$=0.529, P=0.781	$F_{1,26}$=0.049 P=0.827	$F_{6,26}$=0.413 P=0.863
**Anogenital investigation**	$F_{6,26}$ = 0.743, P=0.62	$F_{1,26}$ = 2.438, P=0.131	**$F_{1,26}$ = 9.42, P=0.005**	$F_{6,26}$ = 0.988, P=0.453	$F_{6,26}$=1.133, P=0.371	$F_{1,26}$=1.484 P=0.234	$F_{6,26}$=0.405 P=0.869
**Aggression**	$F_{6,26}$ = 0.547, P=0.767	**$F_{1,26}$ = 12.617, P=0.001**	$F_{1,26}$ = 0.618, P=0.439	$F_{6,26}$ = 0.606, P=0.723	$F_{6,26}$=0.417, P=0.861	$F_{1,26}$=0.15 P=0.702	$F_{6,26}$=0.461 P=0.831
**Grooming**	$F_{6,26}$ = 0.742, P=0.621	**$F_{1,26}$ = 35.59, P<0.001**	$F_{1,26}$ = 3.791, P=0.062	$F_{6,26}$ = 0.524, P=0.785	$F_{6,26}$=0.775, P=0.597	**$F_{1,26}$=7.198 P=0.013**	$F_{6,26}$=0.252 P=0.954
**Allo-grooming**	$F_{6,26}$ = 0.22, P=0.967	$F_{1,26}$ = 739, P=0.398	**$F_{1,26}$ = 4.871, P=0.036**	$F_{6,26}$ = 0.265, P=0.949	$F_{6,26}$=0.579, P=0.743	$F_{1,26}$=4.023 P=0.055	$F_{6,26}$=1.131 P=0.372
**Nesting**	**$F_{6,26}$ = 3.586, P=0.01**	$F_{1,26}$ = 2.331, P=0.139	$F_{1,26}$ = 0.001, P=0.974	$F_{6,26}$ = 0.882, P=0.522	$F_{6,26}$=0.991, P=0.452	$F_{1,26}$=3.292 P=0.081	$F_{6,26}$=1.041 P=0.438
**Burrowing**	**$F_{6,26}$ = 2.706, P=0.035**	$F_{1,26}$ = 0.396, P=0.535	$F_{1,26}$ = 1.186, P=0.286	$F_{6,26}$ = 0.443, P=0.843	$F_{6,26}$=0.383, P=0.883	$F_{1,26}$=3.563 P=0.07	$F_{6,26}$=0.679 P=0.668
**Scrape digging**	$F_{6,26}$ = 0.543, P=0.771	**$F_{1,26}$ = 6.879, P=0.014**	$F_{1,26}$ = 0.409, P=0.523	$F_{6,26}$ = 0.693, P=0.656	$F_{6,26}$=0.551, P=0.765	**$F_{1,26}$=4.233 P=0.049**	$F_{6,26}$=0.434 P=0.849

**Feeding**	$F_{6,26} = 0.776$, P=0.596	$F_{1,26} = 0.633$, P=0.433	**$F_{1, 26} = 14.84$, P<0.001**	$F_{6,26} = 0.145$, P=0.988	$F_{6,26}=0.765$, P=0.604	**$F_{1,26}=10.745$ P=0.003**	$F_{6,26}=0.171$ P=0.982
**Drinking**	$F_{6,26} = 0.935$, P=0.487	**$F_{1,26} = 7.992$, P=0.009**	$F_{1, 26} = 0.195$, P=0.663	$F_{6,26} = 0.393$, P=0.877	$F_{6,26}=0.755$, P=0.612	$F_{1,26}=2.922$ P=0.099	$F_{6,26}=0.179$ P=0.980
**Clustering**	$F_{6,26} = 0.742$, P=0.621	$F_{1,26} = 2.839$, P=0.104	$F_{1, 26} = 0.046$, P=0.831	$F_{6,26} = 0.759$, P=0.608	$F_{6,26}=1.135$, P=0.37	$F_{1,26}=0.392$ P=0.537	$F_{6,26}=0.309$ P=0.926
**Tube running**	$F_{6,26} = 0.675$, P=0.671	**$F_{1,26} = 14.991$, P<0.001**	$F_{1, 26} = 0.53$, P=0.473	$F_{6,26} = 0.296$, P=0.933	$F_{6,26}=0.325$, P=0.918	**$F_{1,26}=5.819$ P=0.023**	$F_{6,26}=0.187$ P=0.978
